# Electrodepositing Ag on Anodized Stainless Steel for Enhanced Antibacterial Properties and Corrosion Resistance

**DOI:** 10.3390/jfb16010019

**Published:** 2025-01-09

**Authors:** Yi Shao, Yue Jiang, Yongfeng Wang, Qiangsheng Dong, Cheng Wang, Yan Wang, Feng Xue, Chenglin Chu, Jing Bai

**Affiliations:** 1 Jiangsu Key Laboratory for Advanced Metallic Materials, School of Materials Science and Engineering, Southeast University, Nanjing 211189, China; sy@i-bmd.org (Y.S.); sadhu_jiang@126.com (Y.J.); cheng.wang@seu.edu.cn (C.W.); xuefeng@seu.edu.cn (F.X.); 2 Institute of Biomedical Devices (Suzhou), Southeast University, Suzhou 215163, China; yfwang0319@163.com (Y.W.); wangyan@i-bmd.org (Y.W.); 3 Jiangsu Key Laboratory of Advanced Structural Materials and Application Technology, School of Materials Science and Engineering, Nanjing Institute of Technology, Nanjing 211167, China; qsdong@njit.edu.cn

**Keywords:** stainless steel, anodization, corrosion resistance, antibacterial property

## Abstract

Antibacterial stainless steels have been widely used in biomedicine, food, and water treatment. However, the current antibacterial stainless steels face challenges in balancing corrosion resistance and antibacterial effectiveness, limiting their application range and lifespan. In this study, an oxide layer sealed with antibacterial Ag particles was constructed on the surface of 304 stainless steel through anodizing and electrodeposition, and the process parameters were optimized for achieving long-term antibacterial properties. The electrochemical tests demonstrated that the composite coating effectively enhanced the corrosion resistance of 304 stainless steel. The X-ray photoelectron spectroscopy analysis revealed the close binding mechanism between the Ag particles and the micropores in the oxide layer. Furthermore, the antibacterial stainless steel has an antibacterial rate of 99% against *Escherichia coli* (*E. coli*) and good biocompatibility. This study provides an effective approach for designing efficient, stable, and safe antibacterial stainless steel.

## 1. Introduction

Stainless steel is one of the most widely used metal materials due to its outstanding corrosion resistance, mechanical properties, and machinability, making it favored in various fields [1,2]. However, in specific areas such as medical devices and kitchenware, bacterial contamination occurs on conventional stainless steels, causing cross-infection risk and posing a serious threat to human health. Therefore, antibacterial stainless steels have garnered widespread attention and become a crucial topic of current research.

Various types of antibacterial stainless steels have been developed, such as surface-treated antibacterial stainless steel, alloy-based antibacterial stainless steel, etc. [2,3,4]. The methods for preparing surface-treated antibacterial stainless steel mainly include surface coating [5,6], ion implantation [7,8], magnetron sputtering [9,10], and electrodeposition [11,12,13]. Although these antibacterial stainless steels have achieved certain success in antibacterial effects, there are many disadvantages such as high cost and poor corrosion resistance. Due to the frequent exposure to corrosive environments, antibacterial stainless steel with poor corrosion resistance cannot be used in serious corrosion situations. In addition, antibacterial agents are introduced into stainless steel for enhanced antibacterial properties, but alter material structures, thereby affecting its corrosion resistance and other aspects of performance. Currently, the process costs for most antibacterial stainless steels are relatively high, such as alloy-based stainless steel requiring a large number of antibacterial elements, while surface-treated types equipment is complex and costly, making it difficult to achieve large-scale production. Therefore, it is particularly important to develop a low-cost antibacterial stainless steel with good antibacterial properties and excellent corrosion resistance.

Anodizing is a well-established technique for preparing porous oxide films on metal surfaces [11,14,15]. The parameters such as pore size, depth, and porosity can be finely controlled by adjusting the electrolyte composition and relevant oxidation processes. Consequently, a uniformly ordered nanoporous film structure can be formed on metal surfaces, significantly enhancing the corrosion resistance and wear resistance of metal materials [16]. More importantly, the unique potential of the nanoporous film structure provides a carrier for antibacterial elements filling, successfully creating metal materials with antibacterial properties. In comparison to ion implantation surface modification, anodizing provides a deeper depth for the deposition of antibacterial elements [17], revealing its enormous potential for application in the preparation of special performance stainless steels.

In this study, the 304 stainless steels were selected as the substrate, one of the most widely used stainless steel brands in the medical device and food industry, and antibacterial stainless steel was successfully prepared using the anodizing and electrodeposition methods. By optimizing the processing technology, we obtained a novel antibacterial stainless steel with excellent antibacterial and corrosion resistance based on low production costs. Therefore, this study provides a new perspective for the preparation of novel antibacterial stainless steel.

## 2. Material and Methods

### 2.1. Materials Preparation

Commercial 304 austenitic stainless steel (0.04% C, 1.02% Mn, 0.03% P, 0.01% S, 0.44% Si, 18.4% Cr, 7.93% Ni) (2 cm × 2 cm × 1 mm) (Zhejiang Qingshan Iron and Steel Co. Ltd, Lishui, China) was used as the substrate. The stainless steel samples were mechanically polished then cleaned in an ultrasonic cleaner (PS-120A, JieKang Ultrasonic Equipment Co., Ltd, Dongguan, China.) after the degreasing treatment (50 g/L NaOH, 40 g/L Na_2_CO_3_, 30 g/L Na_2_PO_4_, 5 g/L Na_2_SiO_3_) to ensure a clean surface. An electropolishing was performed for samples in the electrolyte (600 mL/L H_3_PO_4_, 300 mL/L H_2_SO_4_, 50 mL/L glycerol, 5 mL/L saccharin) at 50 °C. The stainless steel sheet was used as the anode, and the graphite sheet as the cathode, with a cathode-to-anode area ratio of 1.5:1 and an anode–cathode distance of 5 cm. The electrolyte was stirred using a magnetic stirrer (RCT basic, IKA, Staufen, Germany) at a stirring rate of 20 rpm. The electropolishing was carried out for 5 min using a fully controlled DC stabilized power supply (MS1003D, MaiSheng Power Technology Co., Ltd, Shenzhen, China) at a current density of 30 A/dm^2^.

### 2.2. Antibacterial Surface Modification

A mixed solution of phosphoric acid (H_3_PO_4_) and ethylene glycol (EG) was used as the electrolyte for anodization. Pre-treated stainless steel samples were placed in the electrolytic cell, with the samples serving as the anode, and graphite serving as the cathode. The cathode-to-anode area ratio was maintained at 1.5:1, and the cathode–anode distance was set as 5 cm. Anodization was conducted at 0 °C, with an applied voltage of 15 V for 1 h. The antibacterial film containing silver was further prepared by electrochemical deposition using anodic oxidation film of stainless steel as carrier. The alternating current deposition was conducted using a voltage-controlled mode, with the addition of MgSO_4_ as an auxiliary during the deposition process [18]. The process parameters for the electrolyte are as follows: the electrolyte consisted of 2 g/L of silver nitrate, 20 g/L of sulfuric acid, and 12 g/L of magnesium sulfate, with the temperature controlled at 25 °C.

### 2.3. Surface Microstructure Characterization

The surface microstructure of the stainless steel samples with the anodized oxide film and antibacterial layer were observed by field emission scanning electron microscope (SEM) (Sirion200, FEI, Hillsboro, OR, USA). The X-ray photoelectron spectroscopy (XPS) (K-Alpha+, Thermo Fisher Scientific, Waltham, MA, USA) was used to determine the elemental composition and chemical states of surface films on the stainless steel. During XPS analysis, the surface of the antibacterial film was etched at 0.25 nm/s by argon ion sputtering, and the elements in different positions in the film were quantitatively and chemically analyzed by the atomic sensitivity factor method.

### 2.4. Wear Resistance Tests

The wear resistance of the antibacterial layer on the stainless steel surface was assessed using the rubber eraser abrasion tester (DDC-NM290, Dongguan Zhongyi Chuangtian Instrument Equipment Co., Ltd, Dongguan, China.), with untreated stainless steel samples as the control. The rubber wheel used in this study has a shore hardness of 60 degrees (60 HA) and the test stroke is 10 mm, and one round trip is one cycle. The tester was set at a speed of 50 rpm, and the surfaces were worn with a rubber eraser under a load of 4.9 N for 1000 reciprocating abrasions. The weight loss was measured by a precision analytical balance to calculate wear resistance.

### 2.5. Corrosion Resistance Evaluation

The ParStat4000 electrochemical workstation (Ametek, Inc, Berwyn, PA, USA) was used to evaluate the corrosion resistance of the uncoated and coated stainless steel at room temperature. A 3.5 wt% NaCl solution was utilized as the corrosive medium, and a three-electrode cell was employed in which the stainless steel sample was the working electrode, a platinum sheet electrode was the auxiliary electrode, and a saturated calomel electrode (SCE) was the reference electrode. The coated samples with an exposed area of 1 cm^2^ were immersed in the corrosive medium for 30 min to stabilize the open-circuit potential. Electrochemical impedance spectroscopy (EIS) plots were collected at a frequency range of 10 mHz to 100 kHz. Potential polarization curves were obtained at a potential range of −0.5 V to 0.5 V and a scanning rate of 1 mV/s to fully capture the electrochemical behavior of the tested samples and maintain the validity of the Tafel region for accurate corrosion parameter determination.

### 2.6. Antibacterial Performance Tests

*Escherichia coli* (*E. coli*) and *Staphylococcus aureus* (*S. aureus*) were selected as the test bacterial strains to evaluate the antibacterial effect of the stainless steel surface according to ISO 22196-2011 standard [19]. *E. coli* (CICC 10899) and *S. aureus* (CICC 10384) were purchased from the China Center of Industrial Culture Collection (CICC). Untreated stainless steel was used as the control group. A droplet of 0.4 mL bacterial solution with a concentration of 2.5 × 10^5^~10 × 10^5^ CFU/mL was placed on the surface of a 50 mm × 50 mm × 1 mm test piece and covered with polyethylene (PE) film. The samples were incubated at 37 °C for 48 h, followed by colony counting. Each group was performed in triplicate for statistical reliability.

### 2.7. Cell Activity Assay

Live/Dead Cell Staining: Normal Human Dermal Fibroblasts (NHDFs) were cultured in a complete culture medium containing 10% fetal bovine serum and antibiotics (100 U/mL penicillin, 100 µg/mL streptomycin) at 37 °C in a 5% CO_2_ humidified incubator. The experimental group was treated with 100 µL of different concentrations of a new type of antibacterial stainless steel (NASS) extract in the culture medium, whereas the positive control group was treated with 100 µL culture medium containing DMSO. The cells were cultured for 24 h. Subsequently, following the instructions of the Live/Dead Cell Imaging Kit (C2015M, Beyotime, Shanghai, China), the culture medium was removed, and the cells were washed once with PBS. Then, 100 µL of Calcein AM/PI detection working solution was added to the cells, and they were incubated in the dark at 37 °C for 30 min. Subsequently, images were captured using an inverted fluorescence microscope (IX83, Olympus, Tokyo, Japan). Live cells emitted green fluorescence, while dead cells emitted red fluorescence. Each group was performed in triplicate for statistical reliability.

MTT assay: After culturing NHDF cells in a 37 °C, 5% CO_2_ humidified incubator for 24 h, different concentrations of a new antibacterial stainless steel (NASS) extract were added to the experimental group. Subsequently, 100 µL of 0.5 mg/mL MTT solution (CT0025, Leagene, Beijing, China) was added to each well after another 24 h of incubation. The cells were further incubated at 37 °C in the dark for 4 h, following which the absorbance at 490 nm (A490nm) was measured. Each group was conducted with 5 replicates to ensure statistical reliability.

### 2.8. Statistical Analysis

Statistical data were analyzed using GraphPad Prism 8 software (v8.4.2), and the significance was analyzed using the ordinary one-way ANOVA method. Significance was denoted by letters, where the same letter indicates no significance, while different letters indicate significant differences. All data were presented as mean ± SD (n ≥ 3).

## 3. Results and Discussion

### 3.1. Preparation and Characterization of Anodic Oxidation Layer on Stainless Steel

In order to obtain a uniformly ordered nanoporous oxide film, pre-treated stainless steel samples were conducted using anodic oxidation. The research indicated that anodic oxidation using phosphoric acid (H_3_PO_4_) as an electrolyte can form dense and stable oxide films on stainless steel, thereby improving its corrosion resistance. Additionally, ethylene glycol (EG) as a solvent can protect the stainless steel surface to retard serious anodic oxidation effects for enhanced corrosion resistance [15]. Therefore, a H_3_PO_4_-EG electrolyte was used for anodic oxidation and the optimal conditions for anodic oxidation were studied by adjusting the ratio of H_3_PO_4_ and EG.

**Figure 1:**
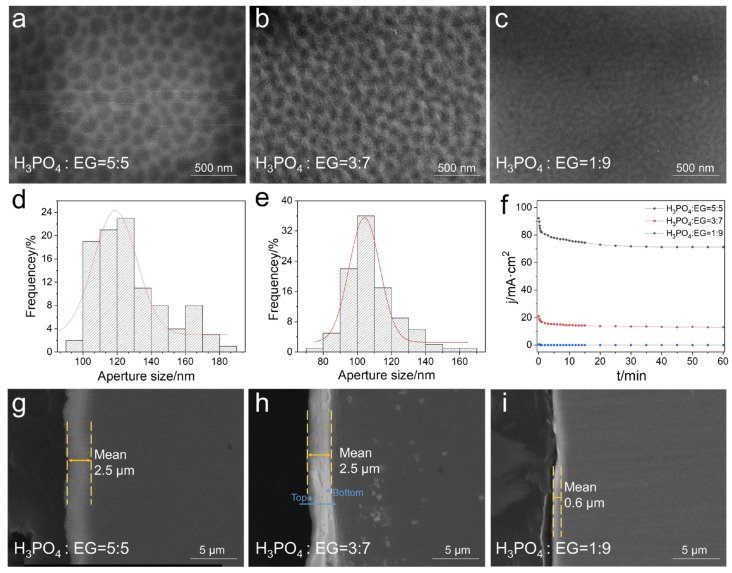
The effects of electrolyte with different phosphoric acid contents on the anodization of stainless steel. (**a**–**c**) Surface morphology of stainless steel after anodization; (**d**–**f**) Relationship curve between anodization current density and time. The red line in (**d**,**e**) represents the fitted curve. (**g**–**i**) Cross-sectional morphology of stainless steel after anodization. * in (**h**) represents the top and bottom positions used to measure the EDS spectrum of the anodized stainless steel film.

The results indicated that the ratio of H_3_PO_4_ and EG affected anodic oxidation effects on the surface of stainless steel (Figure 1). When the ratio of H_3_PO_4_:EG was 5:5, the stainless steel surface formed closely packed small pores with an average pore diameter of 118 nm, exhibiting relatively regular polygonal shapes, poor orderliness, and continuous pore phenomena. The average thickness of the membrane reached 2.5 μm (Figure 1a,d,g). At the ratio of H_3_PO_4_:EG was 3:7, the average pore diameter decreased to 105 nm, with pores appearing as regular hexagons, demonstrating good orderliness and a more uniform distribution. The membrane also exhibited the highest density, and the average thickness of the membrane reached 2.5 μm (Figure 1b,e,h). When the ratio of H_3_PO_4_ decreased to 1:9 of H_3_PO_4_:EG, the surface did not form a micro-nanopore structure but appeared as irregular striped patterns, with an average membrane thickness of only 0.6 μm (Figure 1c,i).

When the ratio of H_3_PO_4_ to EG is 5:5 and 3:7, the current density versus time curve shows a significant inflection point, initially decreasing linearly due to the formation of a barrier layer. As the reaction progresses, the barrier layer gradually dissolves, leading to the formation of a porous film structure on the stainless steel surface. At this point, the current density stabilizes or tends to slowly increase. Since the current density is higher at a ratio of 5:5, the reaction is more intense, resulting in nanopore structures with slightly larger pore sizes compared to a ratio of 3:7, with a more disordered pore distribution. Additionally, due to the higher current density, more heat is released during the reaction, intensifying the dissolution process and resulting in shallower pores. When the ratio is 1:9, it is speculated that the current density is too low to enable the formation of nanopore structures on the surface (Figure 1f). Therefore, in order to achieve the optimal effect of anodized porous membrane-loaded antibacterial agents, an electrolyte with a ratio of H_3_PO_4_:EG of 3:7 was used.

**Figure 2:**
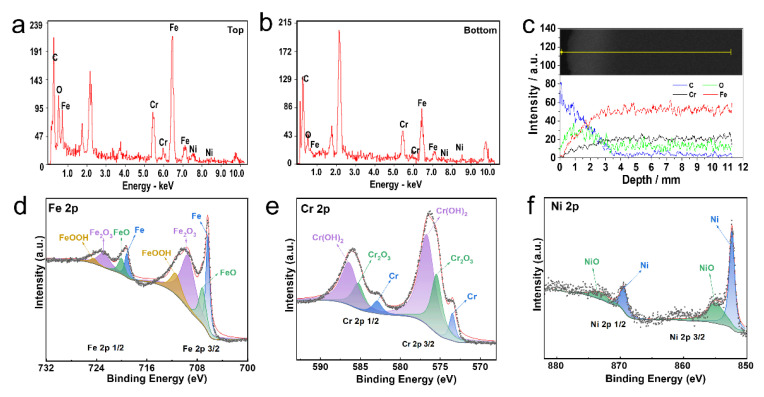
EDS spectrum and XPS spectrum of stainless steel anodized film. EDS spectra of the top (**a**), bottom (**b**), and section line scan (**c**) of the anodic oxide film. The yellow line in (**c**) represents the scanning line for EDS monitoring. (**d**–**f**) XPS spectrum of anodized stainless steel film. The black points represent raw data from XPS measurement, and the red lines represent the fitting data for XPS analysis.

Surface analysis was performed using an energy-dispersive spectrometer (EDS) attached to Sirion200 SEM to investigate the chemical composition of the stainless steel anodic oxidation film (Figure 2). EDS line scan spectra revealed that the oxygen content at the top of the oxide film was significantly higher than in the base material, while the content of metal elements such as Fe, Cr, and Ni was noticeably lower in the oxide film. Particularly, the Fe content exhibited the most significant decrease (Figure 2a–c and Table S1). These results indicated that the nanoporous anodic oxidation film formed by anodic oxidation on the stainless steel surface was mainly composed of oxides of Fe, Cr, and Ni, in which oxides of Fe were dominated. These findings aligned with the mechanism of stainless steel surface anodic oxidation [20].

XPS was performed to clarify the chemical states of O, Fe, Cr, and Ni in the oxide film, as shown in Figure 2d–f. Fe 2p 3/2 can be distinguished to four peaks at 706.7 eV, 707.10 eV, 709.60 eV, and 711.50 eV, which, respectively, represent combined states of Fe in FeO, metallic Fe, Fe_2_O_3_, and FeOOH after deconvolution (Figure 2d) [21,22]. In addition, four deconvoluted peaks are also dissociated in the Fe 2p 1/2 peak. Relatively, Cr 2p 3/2 can be divided into peaks at 573.4 eV, 575.5 eV, and 576.7 eV, which relate to Cr, Cr_2_O_3_, and Cr(OH)_2_ (Figure 2e). According to Ni 2p, Ni 2p predominantly existed in the forms of metallic Ni and NiO (Figure 2f) [23,24]. These findings suggested that the porous film formed on the surface after the anodic oxidation of stainless steel was primarily composed of hydroxides.

**Figure 3:**
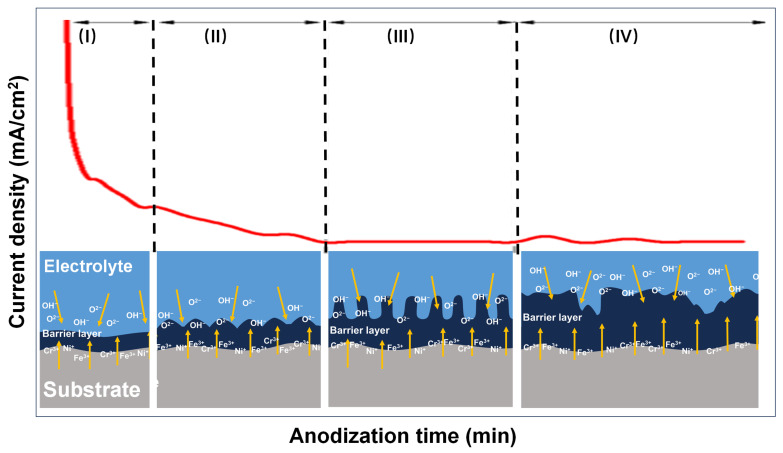
Schematic illustration of the structural changes in the porous film on the surface of stainless steel. The yellow arrows represent the ion migration tracks, and the red curve represents the variation of current density as a function of anodization time. I, II, III and IV represent the anodization stage, respectively.

While anodic oxide films of stainless steel have been prepared by different techniques, many researchers have also studied the formation mechanism of the nanoporous film structure during the anodic oxidation process [11,25,26]. In this study, it was found that in the anodizing process of stainless steel, the curve of current density with time decreased rapidly at the initial stage of the reaction, and then gradually stabilized after falling to the minimum value, and only fluctuated in a small range (Figure 3).

The surface anodic oxide film of stainless steel was formed at the interface between the electrolyte and the stainless steel substrate, where O^2−^ and OH^-^ migrated from the electrolyte to the interface, while metal ions such as Fe^2+^/Fe^3+^, Cr^3+^/Cr^6+^, and Ni^2+^ migrated from the substrate to the interface. The porous film formed on the surface of stainless steel after anodizing is mainly composed of hydroxides such as FeOOH, Cr(OH)_3_, NiOOH, and metal oxides such as Fe_2_O_3_, Cr_2_O_3_, and NiO. It can be inferred that the following reactions occurred at the anode:

2H_2_O → O_2_ + 4H^+^ + 4e^−^

Cr^3+^ + 3OH^−^ = Cr(OH)_3_

*x*M (Fe, Cr, Ni) + O_2_ → M*_x_*O*_y_*(Fe_2_O_3_, Fe_3_O_4_, Cr_2_O_3_, CrO_3_, NiO)

As shown in Figure 3, the formation mechanism of the porous film on the surface of stainless steel can be roughly divided into four stages.

(I) During the anodizing process under constant voltage, a dense and uniform oxide film, also known as the barrier layer, was rapidly formed on the surface of the stainless steel. At this stage, the current density rapidly decreased.
(II) The uneven oxide film on stainless steel caused the change in surface roughness, and the electric field distribution became extremely disordered. Local electric field concentration occurred at the depressions in the oxide layer, resulting in accelerated dissolution of the oxide film and the formation of a large number of nanoscale micropores.
(III) When the formation rate at the film bottom was balanced with the dissolution rate at the film top, the growth of the porous layer entered a stable stage. At this stage, the porous layer exhibited a uniformly distributed pore structure, and the current density stabilized.
(IV) With further extension of time, the balance between the dissolution and the formation of the oxide layer was disrupted. At this stage, the dissolution rate at the edge of the pores increased, exceeding the rate of formation of the bottom oxide layer. The nanoporous structure began to dissolve, resulting in a reduction in depth. At the same time, pore edge collapse occurred in some areas, and the nanostructures began to be destroyed.

Nanoporous structures were prepared on the surface of stainless steel by anodizing, and the pore size and depth could be controlled by the process parameters, thus providing a carrier and space for the filling antibacterial elements, which was a novel method for preparing surface-modified antibacterial stainless steel [17]. Although nanoporous oxide films with ideal thickness and excellent properties have been prepared through anodic oxidation, the commonly used oxidation processes were sulfuric acid–chromic acid or phosphoric acid–chromic acid systems [11]. This type of electrolyte system largely produced hexavalent chromium ions, posing severe risks to human health and causing persistent environmental pollution [27,28,29,30]. Studies have shown that an appropriate amount of trivalent chromium not only is not harmful to human health but also has immune-enhancing functions, and is therefore present in many food and supplement products [31,32,33].

This study innovatively selected an H_3_PO_4_-EG electrolyte system to prepare an anodic oxide film on stainless steel, which mainly consisted of hydroxides such as FeOOH and Cr(OH)_3_, and oxides such as Fe_2_O_3_ and Cr_2_O_3_, with chromium existing in the form of trivalent chromium ions (Figure 2d–f). Therefore, the electrolyte system in this study was more environmentally friendly compared to conventional electrolyte systems.

### 3.2. Preparation and Characterization of Ag Antibacterial Layer on Stainless Steel

**Figure 4:**
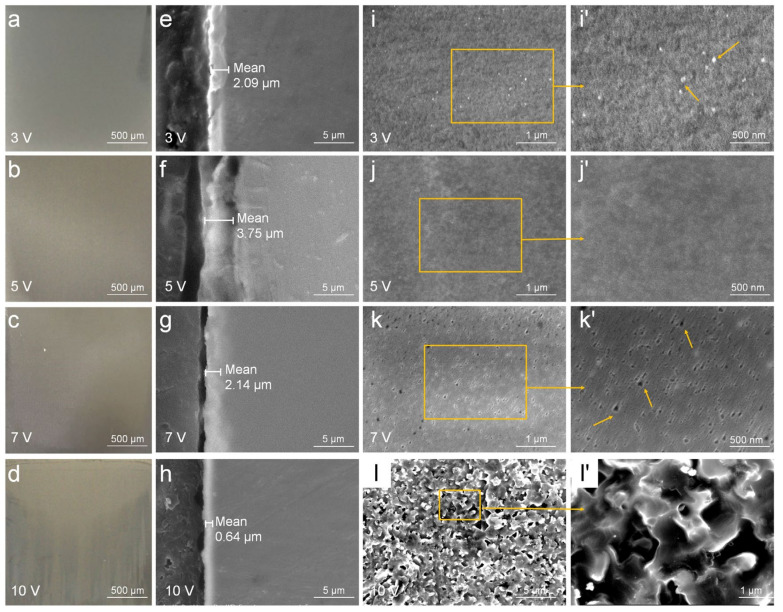
The morphologies of Ag-containing antibacterial layer on stainless steel surface. (**a**–**d**) Appearance of stainless steel samples after electrodeposition at different voltages. (**e**–**h**) The cross-section morphologies and (**i**–**l′**) the surface morphologies were observed by SEM. (**i**′–**l**′) is the magnification of the yellow box in (**i**–**l**), respectively. The deposition time was 2 min and the deposition voltages were 3 V, 5 V, 7 V, and 10 V, respectively.

In order to enhance the adhesion of the metal coating, an Ag-containing antibacterial layer was prepared on the surface of anodized stainless steel using the alternating current deposition method. In comparison to direct current deposition, the pulse interval during alternating current deposition constrained the extent of crystal growth, thereby reducing the possibility of forming large coarse crystals. It is important to note that the deposition time should not be too short or too long, as either scenario may result in antibacterial particles not being fully embedded within the porous film or give rise to severe corrosion on the surface of the stainless steel (Figure 4a–d).

The effect of different deposition voltages on the microstructure of the antibacterial layer was studied under the deposition time of 2 min (Figure 4e–l′). The results indicated that under an alternating current voltage of 3 V, the porous layer was covered with a uniformly thick deposition layer of approximately 2.09 μm (Figure 4e). However, the filling degree within the porous layer varied, and local areas exhibited particle aggregation, as indicated by the arrows (Figure 4i,i′). The thickness of the deposited layer on the stainless steel surface exhibited a certain degree of thinning relative to the thickness of the porous film after anodic oxidation. This is because during the initial stage of AC electric deposition, the reduction reaction of hydrogen ions caused a certain degree damage to the barrier layer of the oxide film, resulting in a thinner barrier layer of the oxide film. When the voltage was increased to 5 V, the density and uniformity of the deposition layer improved along with the deposited material filling the porous structure, resulting in a thickness increase to approximately 3.75 μm, and the microporous structure on the stainless steel anodized film nearly disappeared (Figure 4f,j,j′). Although the barrier layer initially experienced a certain degree of thinning, following the decrease in thickness of the barrier layer, the deposition reaction of Ag accelerated. Under higher voltage, it continuously reduced and deposited onto the stainless steel surface, resulting in a uniformly increasing thickness of the deposited layer. When the voltage was 7 V, the surface of the deposition layer displayed noticeable unevenness and defects, with numerous irregular small micropores, as indicated by the arrows, and the thickness decreased to 2.14 μm (Figure 4g,k,k′). Under a voltage of 10 V, severe corrosion occurred on the antibacterial stainless steel surface, with large areas of the antibacterial layer peeling off, rendering it difficult to obtain a smooth surface, and the antibacterial layer essentially disappeared (Figure 4h,l,l′). These results indicated that excessively high voltage caused the antibacterial ions to be reduced too quickly on the stainless steel surface, leading to local peeling or breakdown of the deposition layer, thus affecting the integrity and stability of the antibacterial layer.

**Figure 5:**
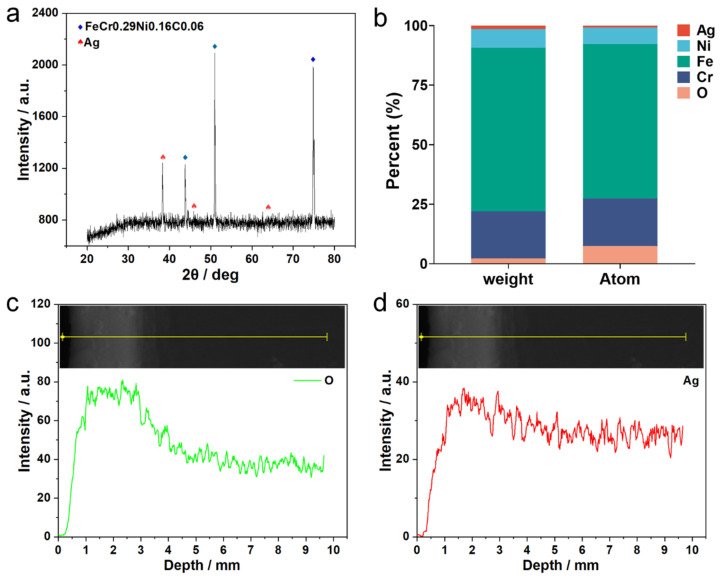
Composition analysis of antibacterial film on stainless steel surface. (**a**) XRD spectrum of antibacterial film surface; (**b**) Distribution of surface elements on antibacterial film; (**c**,**d**) EDS spectrum of cross-sectional line scan of antibacterial film. The yellow lines represent the scanning lines for EDS monitoring, the green line displays the distribution of O element, and the red line in (**d**) displays the distribution of Ag element.

The elemental composition and chemical state of the antibacterial film on the stainless steel surface were further analyzed (Figure 5). The XRD spectra showed that in addition to the basic elements O, Cr, Fe, and Ni of the stainless steel porous oxide film, the antibacterial film also contained a certain amount of Ag (Figure 5a,b). EDS spectra of cross-sectional line scanning revealed a substantial increase in oxygen content relative to the matrix (Figure 5c), which was consistent with the change in elemental content of the porous film carrier on the surface of stainless steel, proving that the carrier was a porous film with metal oxides as its main component. The content of the Ag element also increased to a certain extent compared to the substrate (Figure 5d). Additionally, the XRD phase analysis of the antibacterial layer indicated the presence of diffraction peaks with a relatively high content of Ag.

**Figure 6:**
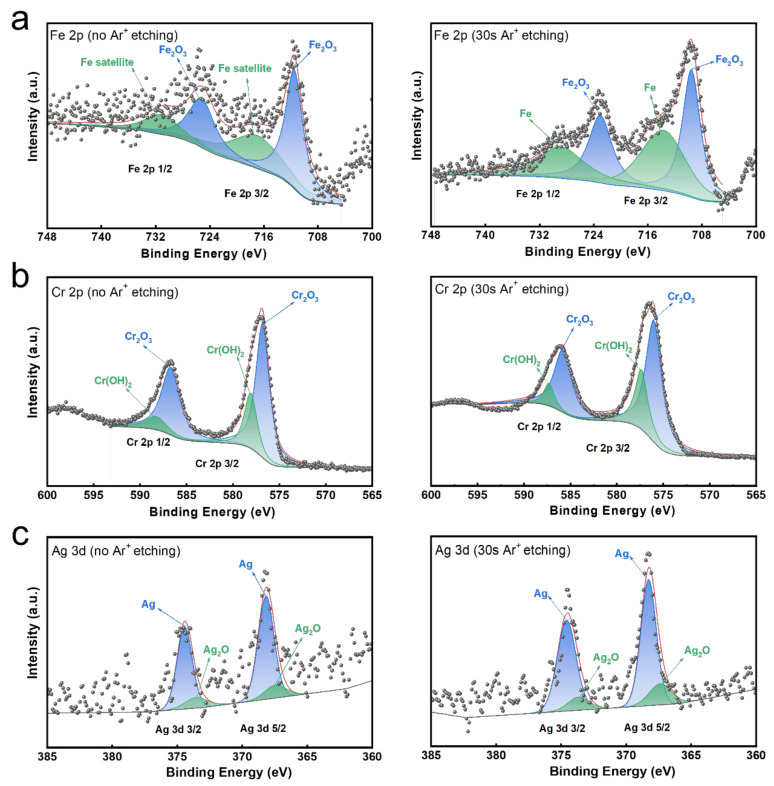
XPS spectrum of Ag-containing antibacterial film on stainless steel surface. (**a**) Fe 2p3; (**b**) Cr 2p3; (**c**) Ag 3d. The black points represent raw data from XPS measurement, and the red lines represent the fitting data for XPS analysis.

Figure 6 displayed the XPS results of Ag-containing antibacterial film on the stainless steel surface. The Fe 2p spectrum is composed of Fe_2_O_3_ peaks and Fe-related satellite peaks (Figure 6a). After 30 s of etching, the Fe-related satellite peaks disappear. The Fe 2p 3/2 spectrum can be dissociated into two peaks of Fe (713.9 eV) and Fe_2_O_3_ (709.6 eV). Whether or not it has been etched, the Cr 2p 3/2 spectrum displays two peaks at 576.80 eV and 578.10 eV, representing Cr_2_O_3_ and Cr(OH)_3_ (Figure 6b). The Ag 3d spectrum showed that the photoelectron peaks of 368.4 eV and 374.27 eV corresponded to Ag and Ag_2_O, and the Ag content at the bottom of the deposition layer was higher than that at the top (Figure 6c), indicating that Ag existed in the form of elemental Ag and its oxide states, and was deposited within the pores of the porous oxide film.

### 3.3. Corrosion Resistance of the Stainless Steel

To assess the practical value of the stainless steel, the wear resistance and corrosion resistance were tested. The results showed that no obvious scratches or spalling occurred in all samples after 1000 reciprocating times, with minimal changes in both mass and color (Table S2). It is proved that the stainless steel surfaces with Ag-containing films exhibit excellent wear resistance.

**Figure 7:**
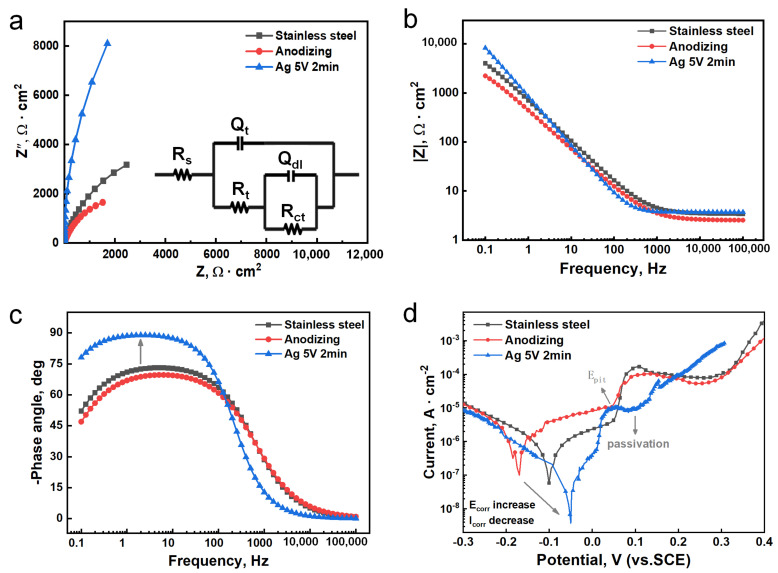
AC impedance spectrum of antibacterial stainless steel at different deposition times. (**a**) Nyquist; (**b**) Bode |Z|; (**c**) Bode-Phase; (**d**) polarization curves.

Then, the corrosion resistance of the Ag-containing films stainless steel was studied by electrochemical impedance spectroscopy (Figure 7). The Nyquist plot clearly showed that the stainless steel with Ag-containing films had the largest impedance arc radius, indicating the best corrosion protection of the film, while the anodized film exhibited the worst corrosion resistance (Figure 7a). From a quantitative perspective, the Bode plot (Figure 7b) showed that the impedance modulus of the stainless steel with Ag-containing films in the low-frequency region (0.1–10 Hz) was significantly higher than the other two samples. Furthermore, the introduction of Ag caused changes in the properties of the anodized film, as compared to the anodized film without Ag (Figure 7c). As indicated by the arrow in Figure 7c, the phase angle for Ag 5V 2min sample approaching 90° reflects enhanced capacitive behavior, which suggests the formation of a dense and stable passive film, effectively insulating the surface and providing strong protection against corrosion.

The potentiodynamic polarization curves of the stainless steel with Ag-containing films in the corrosive medium are shown in Figure 7d. The ordinate of the intersection points of the anodic and cathodic branches was the corrosion potential (E_corr_), reflecting the ease of corrosion reactions in the samples. A higher E_corr_ indicates lower susceptibility to corrosion [34]. The stainless steel sample deposited for 2 min had the highest self-corrosion potential E_corr_ (−0.051 V vs. SCE) in the corrosive medium, while the E_corr_ of untreated and solely anodized stainless steel was −0.101 V and −0.169 V, respectively. This indicates that the stainless steel samples deposited for 2 min through electrochemical deposition had the lowest propensity for spontaneous corrosion reactions, thus demonstrating superior corrosion resistance.

To comprehensively evaluate its corrosion resistance, the self-corrosion current density (I_corr_) was determined by fitting Tafel plots (Table S4). A lower I_corr_ indicates greater stability of the sample in the corrosive environment. After anodizing, the I_corr_ value for stainless steel increased, whereas deposition of Ag resulted in a significant decrease in I_corr_, indicating that Ag deposition enhanced the corrosion resistance of the anodized stainless steel surface. These results collectively suggested that anodization reduced the corrosion resistance of stainless steel, while electrochemical deposition of Ag significantly enhanced its corrosion resistance.

### 3.4. Antibacterial Properties and Biocompatibility of the Stainless Steel

**Figure 8:**
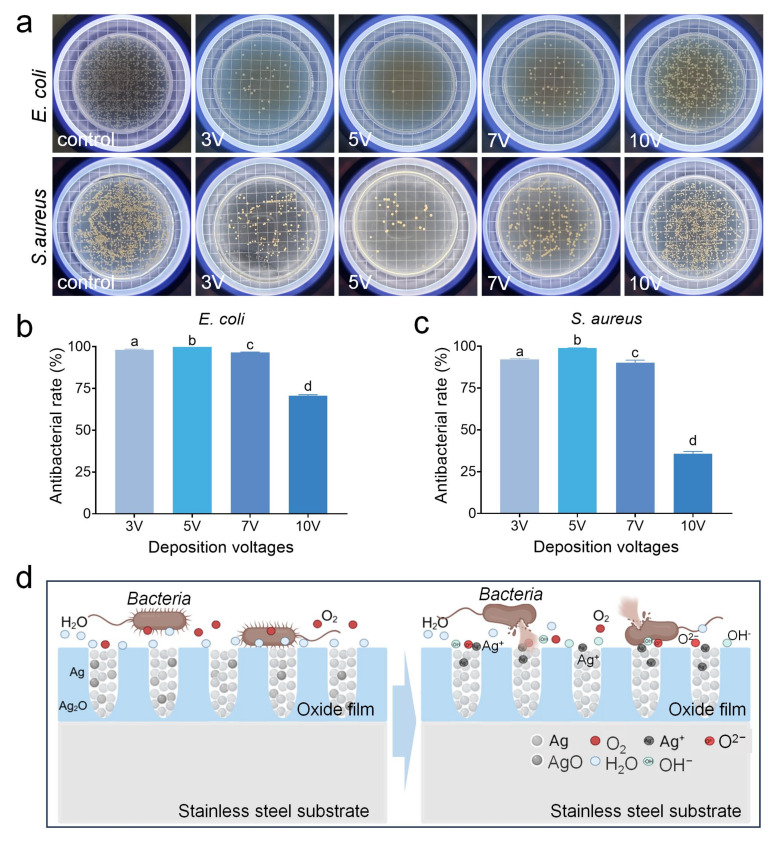
The antibacterial properties of antibacterial stainless steel under different processes. (**a**) Typical colony counting pictures against *E. coli* and *S. aureus*. (**b**) Antibacterial rate against *E. coli*. (**c**) Antibacterial rate against *S. aureus*. Different letters indicate significant differences between groups. (**d**) Schematic diagram of antibacterial film killing bacteria on the surface of stainless steel.

The antibacterial properties of the new stainless steel were further studied. The results showed that the stainless steel with the Ag-containing antibacterial layer exhibited excellent antibacterial effects (Figure 8). In comparison with the specimens prepared at 3 V-2 min, 7 V-2 min, and 10 V-2 min, the stainless steel with the Ag-containing antibacterial film prepared at 5 V-2 min demonstrated the best antibacterial performance, with an antibacterial rate exceeding 99.9% against *E. coli* and 99.8% against *S. aureus* (Figure 8b,c). This aligns with the observed surface microstructure of the Ag-containing antibacterial film, which displayed corrosion.

The previous results showed that the Ag element in the antibacterial film existed in the form of metallic Ag and AgO after deposition (Figure 6), with AgO having a strong reduction potential. Based on the current antibacterial mechanisms of catalytic reaction, it was speculated that silver oxide (AgO) can activate oxygen in air and water, generating reactive oxygen species (O^2−^) and hydroxyl radicals (-OH). O^2−^ exhibited strong oxidation-reduction capability, while -OH was more chemically reactive (Figure 8d). After being entered into bacterial cells, it can combine with the thiol groups in dehydrogenases, hindering bacterial energy metabolism and placing them in a suppressed state, thereby impeding bacterial proliferation and leading to bacterial death.

**Figure 9:**
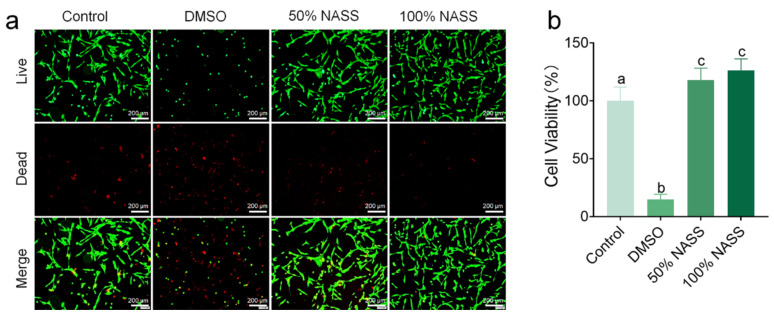
The new antibacterial stainless steel exhibited excellent biocompatibility. (**a**) Live/dead staining of cell morphology after co-culture with the stainless steel extract for 24 h. Live cells were stained as green, and dead cells were stained as red. (**b**) MTT assay used to measure the growth rate of NHDF cells. Control: negative control; DMSO: positive control; NASS: New Antibacterial Stainless Steel. n = 5, different letters denote significant differences between the groups.

The staining of live and dead cells of the new antibacterial stainless steel (NASS) showed that the control group exhibited normal cell development, whereas the positive control DMSO group showed extensive cell death with only a few cells surviving (Figure 9). Interestingly, the new antibacterial stainless steel extracts prepared at 5 V-2 min were co-cultured with NADH cells, the new antibacterial stainless steel extracts prepared at 5 V-2 min were co-cultured with NHDF cells, and the cells not only developed normally, but also showed a significant decrease in dead cells, with a noticeable increase in cell density compared to the control group (Figure 9a). It is noteworthy that the performance of the 100% NASS group exceeded that of the 50% NASS group. Furthermore, MTT assay results further confirmed that both the 50% NASS and 100% NASS groups exhibited higher cell viability than the control group, with the cell viability even surpassing 100% (Figure 9b). These results collectively demonstrated the excellent biocompatibility of the new antibacterial stainless steel, suggesting promising applications in the medical device field.

## 4. Conclusions

In conclusion, this study utilized 304 stainless steels as the substrate and selected a more environmentally friendly system of phosphoric acid-ethylene glycol electrolytes. Through optimized processing techniques of anodization and electrodeposition, a low-cost antibacterial stainless steel with significantly improved antibacterial and corrosion-resistant properties was prepared. Ag-containing film improved the E_corr_ and decreased the I_corr_ of the stainless steel substrate, and exhibited a higher impedance modulus, revealing enhanced corrosion resistance. Future work will include experiments in different media with varying bacterial concentrations to further evaluate the long-term corrosion resistance and antibacterial properties of the materials. Additionally, it demonstrated excellent biocompatibility, making it a potential candidate for medical applications in the future.

## Data Availability

Dataset available on request from the authors.

## Supplementary Materials

The following supporting information can be downloaded at: https://www.mdpi.com/article/10.3390/jfb16010019/s1. Table S1: Element content of different oxide layers on stainless steel anodized film. Table S2: Weight of stainless steel before and after rubbing. Table S3: Equivalent circuit component parameters of AC impedance spectrum. Table S4: Fitting results of antibacterial stainless steel polarization curves under different deposition times.
