# Supplementary material for: Electrodepositing Ag on Anodized Stainless Steel for Enhanced Antibacterial Properties and Corrosion Resistance
Source: J Funct Biomater. 2025 Jan 9;16(1):19. doi: 10.3390/jfb16010019 (PMC11765652; doi:10.3390/jfb16010019)
Supplement: Supplementary file 1 [file jfb-16-00019-s001.zip › jfb-3371460-supplementary.pdf]

Supplementary materials

Electrodepositing Ag on Anodized Stainless Steel for Enhanced Antibacterial Properties and Corrosion Resistance

Yi Shao [1,2], Yue Jiang [1,2], Yongfeng Wang [2], Qiangsheng Dong [3], Cheng Wang [1], Yan Wang [2], Feng Xue [1,2], Chenglin Chu [1,2,*] and Jing Bai [1,2,*]

1 Jiangsu Key Laboratory for Advanced Metallic Materials, School of Materials Science and Engineering, Southeast University, Nanjing, 211189, China; sy@i-bmd.org (Y.S.); sadhu_jiang@126.com (Y.J.);

2 Institute of Biomedical Devices (Suzhou), Southeast University, Suzhou, 215163, China; yfwang0319@163.com (Y.W.); wangyan@i-bmd.org (Y.W.)

3 Jiangsu Key Laboratory of Advanced Structural Materials and Application Technology, School of Materials Science and Engineering, Nanjing Institute of Technology, Nanjing 211167, China; qsdong@njit.edu.cn (Q.D.)

*Correspondence: clchu@seu.edu.cn (C.C.); baijing@seu.edu.cn (J.B.).

Table S1 Element content of different oxide layers on stainless steel anodized film

Element	Stainless steel substrate		Porous oxide film top		Porous oxide film bottom	
	Weight %	Atomic %	Weight %	Atomic %	Weight %	Atomic %
O	1.85	6.11	21.96	49.32	15.06	37.87
Cr	19.97	20.31	13.92	9.63	22.08	17.07
Fe	69.79	66.07	58.21	37.45	57.04	41.06
Ni	8.39	7.51	5.92	3.63	5.84	4.00

Table S2 Weight of stainless steel before and after rubbing

No.	Process parameters	Mass before milling (g)	Mass after milling (g)	Weight loss (g)
$0^{\#}$	Blank	3.7352	3.7349	0.0003
$1^{\#}$	5V 2min	3.5869	3.5868	0.0001
$2^{\#}$	5V 5min	3.7154	3.7151	0.0002
$3^{\#}$	5V 10min	3.6873	3.6871	0.0002

Table S3 Equivalent circuit component parameters of AC impedance spectrum

**Process Parameters**	**R_s (ohms)**	**Q(F)**	**n**	**R_f (ohms)**	**Q(F)**	**R_{ct} (ohms)**	**n**
**Stainless Steel**	3.41	2.998×10^{-4}	0.8351	9644	2.155×10^{-6}	318.3	0.7915
**Anodizing**	3.866	4.562×10^{-4}	0.8212	2495	1.774×10^{-4}	2525	0.7812
**5V 2min**	3.717	1.954×10^{-4}	0.9655	4.02×10^{4}	6.34×10^{-3}	1.253×10^{5}	0.9469

Table S4 Fitting results of antibacterial stainless steel polarization curves under different deposition times

Deposition parameters	E_{corr}(V)	I_{corr}(μA/cm^2)
Stainless Steel	-0.101	0.81
Anodizing	-0.169	1.37
Ag 5V 2min	-0.051	0.25
